# Estimating the Cost of Type 1 Diabetes in the U.S.: A Propensity Score Matching Method

**DOI:** 10.1371/journal.pone.0011501

**Published:** 2010-07-09

**Authors:** Betty Tao, Massimo Pietropaolo, Mark Atkinson, Desmond Schatz, David Taylor

**Affiliations:** 1 Center for Health Research and Policy, CNA, Alexandria, Virginia, United States of America; 2 Departments of Internal Medicine and Pediatrics, University of Michigan, Ann Arbor, Michigan, United States of America; 3 Department of Pathology, University of Florida, Gainesville, Florida, United States of America; 4 Department of Pediatrics, University of Florida, Gainesville, Florida, United States of America; 5 CNA, Alexandria, Virginia, United States of America; Erasmus University Rotterdam, Netherlands

## Abstract

**Background:**

Diabetes costs represent a large burden to both patients and the health care system. However, few studies that examine the economic consequences of diabetes have distinguished between the two major forms, type 1 and type 2 diabetes, despite differences in underlying pathologies. Combining the two diseases implies that there is no difference between the costs of type 1 and type 2 diabetes to a patient. In this study, we examine the costs of type 1 diabetes, which is often overlooked due to the larger population of type 2 patients, and compare them to the estimated costs of diabetes reported in the literature.

**Methodology/Principal Findings:**

Using a nationally representative dataset, we estimate yearly and lifetime medical and indirect costs of type 1 diabetes by implementing a matching method to compare a patient with type 1 diabetes to a similar individual without the disease. We find that each year type 1 diabetes costs this country $14.4 billion (11.5–17.3) in medical costs and lost income. In terms of lost income, type 1 patients incur a disproportionate share of type 1 and type 2 costs. Further, if the disease were eliminated by therapeutic intervention, an estimated $10.6 billion (7.2–14.0) incurred by a new cohort and $422.9 billion (327.2–519.4) incurred by the existing number of type 1 diabetic patients over their lifetime would be avoided.

**Conclusions/Significance:**

We find that the costs attributed to type 1 diabetes are disproportionately higher than the number of type 1 patients compared with type 2 patients, suggesting that combining the two diseases when estimating costs is not appropriate. This study and another recent contribution provides a necessary first step in estimating the substantial costs of type 1 diabetes on the U.S.

## Introduction

Type 1 diabetes (T1D) is an autoimmune disease, often diagnosed early in life, characterized by the destruction of the insulin-secreting β cells in the pancreas. As a consequence, patients become insulin-dependent and must follow a rigid, daily regimen of exogenous insulin replacement. In contrast, type 2 diabetes (T2D) is typically a disease of adulthood (although more cases are now being seen earlier in life), where a relative insulin deficiency arises due to insulin resistance and abnormal pancreatic β cell function [1]. The American Diabetes Association (ADA) estimates that there are 17.9 million individuals diagnosed with diabetes in the U.S. [2], with 5 to 10 percent representing those with T1D [3]. Epidemiologic studies suggest that the incidence rate of T1D has been growing worldwide, especially amongst young children [4].

With the increasing focus on the T2D epidemic, the impact of T1D in this country is often overlooked, particularly from an economic perspective. Many cost-of-illness (COI) studies have been performed on diabetes, but most combine the costs for T1D and T2D over just one year. The Agency for Health Research Quality (AHRQ), using the Medical Expenditures Panel Survey (MEPS), a nationally representative data set [5], reports that $34 billion in health expenditures were related to diabetes in 2005 [6]. A frequently quoted study by the ADA improves upon this number by using the attributable risk methodology to calculate the portion of expenditures on related comorbidities that is due to diabetes [7]. The researchers also include other non-medical costs (i.e. lost work days, bed days, and increased mortality), which are important factors in the cost of diabetes. The study finds that diabetes is responsible for $92 billion in medical expenditures and $40 billion in indirect costs in 2002. An ADA update finds that in 2007 the total cost of diabetes had increased to $174 billion ($155 billion in 2002 dollars), the increase in costs due to increased populations of diabetics [2]. Another recent study projects that the costs of the diabetes epidemic will have risen to $336 billion (2007 dollars) by 2034 as the diabetes patient population exceeds 44 million [8].

These studies do not break down diabetes costs by type in their estimates. Rather, they attempt to apply the proportion of patients with type 1 to obtain the fraction of costs attributed to type 1, an assumption that does not account for the distinct nature and differing disease progressions of the two forms of the disease. Furthermore, there is wide variation in results among the cost studies. For example, a study by the Milken Institute calculates combined T1D and T2D indirect costs to be over $105 billion in 2003 [9]. The discrepancy is due to the inclusion of differing cost components and varying underlying methodologies. Studies that do specifically examine the T1D population concentrate on children, are not nationally representative, are not U.S. based, or only look at medical expenditures [10]–[12]. One recent exception by Dall et al. [13], using medical claims data to identify T1D patients, compares the annual costs of T1D with T2D in the U.S. and finds the costs of T1D to be disproportional to the number of T1D patients compared with the number of T2D patients.

Patients with T1D typically suffer from the disease for a longer period of time. Regular maintenance of T1D requires daily insulin shots and constant monitoring, representing a significant lifelong cost and time requirement. For T1D, these long-term effects are likely to spill over to other aspects of their lives with resulting economic impacts particularly in indirect costs. Milton et al. [12] compile and review studies in the literature addressing the social consequences of T1D. The evidence is mixed. They find that children with T1D are more likely to miss school and that the employment outcomes are worse, but school performance and educational attainment remain unaffected. Providing lifetime cost estimates, as well as annual costs, would allow comparison among diseases that take into account the full economic impact of chronic diseases and facilitate discussions of benefits of possible cures.

To address these deficiencies and provide a comprehensive first step in identifying the annual and lifetime costs of T1D, this study was designed to estimate the direct medical expenditures and indirect costs associated with T1D in the U.S. Together with more traditional biomedical research into the causes and treatments of T1D, this study allows for a comprehensive understanding of the costs and benefits associated with the disease. Doctors, patients, researchers, policy makers, and drug developers can use these results to more fully understand the impact of T1D on individuals and society.

In this paper, we present the results of this analysis that show, as might be expected given the different disease pathologies that underlie the forms of diabetes, that the cost breakdown in T1D is different than T2D, with indirect costs being disproportionately larger for T1D. Furthermore, we show that over a lifetime, the economic costs of T1D are in excess of $10 billion for a new yearly cohort of approximately 30,000 patients.

## Materials and Methods

Using nationally representative data sets, we identify T1D patients and examine direct medical expenditures and indirect costs associated with the disease by matching a T1D patient to a counterpart without diabetes. Since finding an exact match becomes increasingly difficult as more covariates are used, we implement the propensity score matching (PSM) technique first introduced by Rosenbaum and Rubin [14] and frequently used in the program evaluation literature.

Although matching is not new to cost studies, few match on more than simply age, gender, and race. However, to obtain an unbiased and accurate measure of costs attributed to T1D, the matched control individuals must be as similar to the population with T1D as possible so that the only difference between the two can be attributed to T1D. This is not likely to be the case if matching is based on only a few broad demographic variables.

The matching method compares with other cost estimation methods such as those used by the ADA, which require detailed and accurate knowledge of the relationship between diabetes and its comorbidities [2], [7]. Matching methods do not require this knowledge, but there is a trade-off; matching does not work well when important unobservable differences between those with and without the disease exist, as is true for T2D [15]. For example, preferences, life style choices, and genetics are highly correlated with the probability of having T2D. Although MEPS contains some information on exercise habits, diet and the health of family members (except for those living in the same household as relatives) are unknown. However, T1D is a good candidate for matching because unlike T2D, the presence of unobservable factors that affects the probability of having the disease is minimal. In fact, 80 percent of T1D patients do not have a family member with the disease, and behavioral and environmental causes are not definitively known [16], [17].

### Matching

Ideally, one would like to observe the diabetic counterfactual—the life of a patient with diabetes had he not had diabetes—and attribute all differences to diabetes. Of course, the counterfactual does not exist in real life, and therefore the notion of a nondiabetic diabetic-type is pertinent. This is equivalent to the nonsmoker smoker-type, which is used in the smoking literature to determine the cost of smoking [18]–[20]. The nondiabetic diabetic-type does not have T1D, but is the same as a patient with T1D in regards to any differences that distinguish a patient with diabetes from an individual who does not have the disease, including morbidity, mortality, and medical care use. Compared with a fully nondiabetic individual, the nondiabetic diabetic-type individual may use more medical care and experiences a higher mortality rate. On the other hand, compared with an actual patient with T1D, the nondiabetic diabetic-type uses less medical care and has a lower mortality rate, with the difference being attributable to T1D. T1D is not definitively linked with particular behaviors or environmental factors, though research has suggested some possible associations. We include relevant covariates to determine the nondiabetic diabetic-type sample.

Matching each diabetic with a nondiabetic diabetic-type and attributing any differences to T1D requires several assumptions [14]. The first is the Conditional Independence Assumption (CIA), which is stated as follows:

Let *y_0_* be the outcome (such as medical expenditures) for a person without T1D.
Let *y_1_* be the outcome for a patient with T1D.
Let *D* be an indicator for having T1D.

Then,
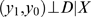
(1)where *X* are the covariates used for matching a patient with T1D to a nondiabetic diabetic-type and ⊥ indicates independence. The outcomes *y_0_* and *y_1_* are independent of whether or not the individual has T1D, after conditioning on all the covariates in *X*. In other words, after matching on *X*, having T1D is the only difference between a T1D patient and his matched counterpart that affects the outcome. The assumption is strong and requires careful consideration in determining the appropriate covariates that affect both the probability of T1D and the outcome of interest.

Including more observable characteristics for matching improves the results of the matching estimator, but makes it increasingly difficult to find an exact match for each individual with diabetes. Matching based on propensity scores addresses this problem by providing a natural weighting scheme for potential matches. The propensity score is the probability of receiving “treatment” conditional on the observable characteristics [14]. In this case “treatment” is the condition of T1D. The probability of having T1D is regressed on a list of covariates through a logit or probit regression. Each individual receives a score based on his predicted probability of having T1D. The individual is then matched pairwise (or some other way) to a nondiabetic by this predicted probability, or score. The propensity score can be written as
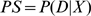
(2)The CIA then becomes 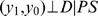. A second assumption of common support is necessary.

It is written as 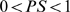, and asserts that there is no perfect predictability of *D* given *X*. Individuals with the same *X* have positive probability of having T1D.

T1D patients are matched using the propensity score to individuals in a control group of those without diabetes. The difference in outcomes between the patients and their matched counterparts is called the average treatment on the treated. It is the portion that can be attributed to T1D. It is written as
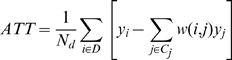
(3)where

*y_i_* is the outcome for an individual with T1D
*y_j_* is the outcome for a nondiabetic diabetic-type
*N_d_* is the number of people with T1D
*C_j_* is the set of matched control individuals for the T1D patients
*w(i,j)* is the weighting function that depends on the PSM method.

The simplest weighting method is a 1 to 1 nearest neighbor matching. The difference between the two groups is then the average difference between the outcome of a T1D patient and his matched counterpart. The average treatment cost difference between a patient with T1D and a person without diabetes is:
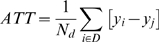
(4)Matching can be done with or without replacement. Replacement decreases the bias by ensuring a better fit, but increases variance since fewer observations are being used. The main analysis in this study uses a logit specification to estimate the T1D propensity score and one-to-one matching with replacement. There are other weighting methods. In testing the sensitivity of the matching method, we use kernel density matching, which uses the all nondiabetics for comparison, but weights their importance by the propensity score. We do not find the results to differ from our main analysis. Standard errors are calculated by bootstrapping with 500 iterations. Using the calculated standard errors, we report 95 percent confidence intervals for all results.

### Matching assessment

A difficult part of the matching process is the determination of appropriate covariates for matching. There is no hard and fast rule for choosing covariates, but there should be a theoretical reason for why the covariate affects both the probability of having T1D and the outcome of interest [21]. Further, it is important that the covariates included are not affected by T1D. For example, say we wished to include family size as an explanatory variable for the likelihood of T1D. Assuming that the parents of T1D patients are less likely to have many children (due to the extra time and resources committed to caring for T1D patients), then the impact of family size on the likelihood of T1D would be confounded since, in this scenario, children with T1D would tend to have fewer siblings. The covariates we include come from the medical literature and vary by the strength of evidence [22], [23].

After matching on propensity scores, the match quality is evaluated by comparing the means of the T1D group and the matched control group. Since matching is made on propensity score, which is weighted naturally by the importance of each variable, the means are not expected to all be exactly the same, but should be close. In general, sample differences in the unmatched case exceed those in the matched case. To quantify the differences between the unmatched and matched samples, we define the following [24], [25]:
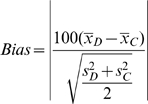
(5)where 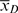 and 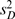 are the sample mean and variance for the T1D sample, and 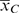 and 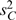 represent the same for the control sample.

### Identifying T1D patients

One likely cause of the small amount of T1D cost studies is the difficulty in distinguishing patients with T1D from those with T2D in secondary data. Most population surveys simply ask whether a person has ever been told by a physician that he has diabetes, and data with detailed medical tests are not nationally representative. One exception is the National Health and Nutrition Examination Survey (NHANES) [26], which administers a c-peptide test for T1D. However, due to the small number of patients with diabetes sampled by NHANES, we use “clinically-derived definitions” to identify individuals with T1D using age of onset, body mass index (BMI), and usage of insulin therapy. The Diabetes Care Survey (DCS), part of MEPS, collects information on individuals who self report having diabetes. However, the DCS does not obtain the age when the individual was first diagnosed with diabetes, but this information can be obtained from the National Health Interview Survey (NHIS) [27]. The MEPS can be linked to the NHIS since MEPS participants consist of a subsample of NHIS participants drawn a year later, allowing variables to be shared between the two data sets. The DCS is available starting from 1999. We pool data from 1999 to 2005, the most recent year with available data, to generate our sample.

Other studies also categorize diabetes based on their patterns of drug use during the study period [10], [28]. For example, Koopman et al. [29] use an age of onset and the exclusive use of insulin for therapy as criteria for categorizing T1D and confirm the validity of these criteria using the NHANES, Prior et al. [30] finds that clinically-derived definitions generally work well to distinguish T1D and T2D. However, categorizing T1D using these qualifications can lead to both over- or under- estimates of the number of T1D patients. A strong indicator of T2D in a child is being overweight or obese [31], [32]. Therefore, we refine the definition of individuals with T1D by eliminating children under the age of 18 who report having diabetes and who are also considered obese. Obesity in children is defined as having a BMI equal to or greater than the 95th percentile by age [33]. The number of T1D patients is underestimated by the number of individuals diagnosed with T1D over the age cutoff we use. This includes those who are misdiagnosed with T2D because of their age. For example, Latent Autoimmune Diabetes of the Adult (LADA) occurs in adults over the age of 30 and is commonly misdiagnosed as T2D in the early stages of the disease. An estimated 10 percent of adults diagnosed with T2D are misdiagnosed and truly have T1D [34], [35]. Since the typical LADA patient is non-obese, we limit the sample to patients whose BMI is 30 or less if they are diagnosed over age 30 [36], [37]. Cusick et al. [38] also employ a weight qualification to obtain their T1D sample. An age 30 cutoff is perhaps the most common in the literature, though studies do use later cutoffs [39] as we do in this study. We limit the sample to include only patients that report currently using insulin. The data set only provides current BMI and therefore a T2D patient diagnosed over the age of 30 who was obese at the time of diagnosis, but who lost weight by the time of the survey, would be categorized as having T1D. In order to avoid underestimating the LADA population, the main analysis uses 45 as the age of diagnosis cut off, which represents 1.1 million T1D patients.

### Estimating yearly costs

Yearly costs are those incurred in an average year between 1999 and 2005. The medical expenditures considered in this study are total expenditures and therefore include self-payments and any third-party payments. In particular, they consist of hospital inpatient, ambulatory visits (consisting of hospital outpatient and physician office-based), emergency room and prescription drug/medical supplies, home health provider, and vision and dental utilization expenditures.

Indirect, or productivity costs, are not as straightforward to measure, and income is frequently used to measure productivity losses due to illness [7]. MEPS divides income evenly between the adults in the household, unless the share of total income by each adult is obtained. To be consistent, we split income evenly between spouses for all applicable cases in our sample. Therefore, even if one member of a family does not generate a market income, he may be coded as having half his spouse's income. A nonworking spouse would therefore be assigned an income, though it possibly is not reflective of his or her productivity. Additionally, Brouwer et al. [40] note that income may not completely reflect productivity due to social benefits and presenteeism (reduced performance when at work). To address these issues, we consider additional productivity measures such as total household income, lost work days, bed days and missed school days. To prevent double-counting, these measures should not be added to income loss; we list them separately.

To estimate productivity losses attributed to early mortality caused by T1D, we compute the present value of future productivity (PVFP) [2]. We estimate the PVFP for those who died by summing expected earnings every year by age and discounting by a three percent discount rate [41]. To estimate the expected earnings profile for each individual, we use the income distribution by age of the current patients in the sample, accounting for the probability of dying every year. To determine the expected number of deaths of T1D patients, we use the mortality rates from the literature and the current number of patients [42], [43]. We assess the cost attributed to T1D by performing the same PVFP calculation on the matched control sample using national mortality rates and attributing the difference to the disease [44].

Propensity score matching ensures that we account for all observed variables in determining the control sample and the subsequent effects on outcomes. However, the results will be biased if an unknown, unobserved factor affects whether an individual has T1D and also the outcomes we observe. A Rosenbaum bounds test can be performed to verify that our findings are not driven by a potential omitted variable [25], [45]. The existence of this unobserved variable and its omission from the model would bias the estimated effects of T1D. We find that our results are insensitive to a bias unless an unobserved factor more than doubled the odds of having T1D, allowing us to be reasonably certain that our methods accurately estimate the effect of T1D.

### Estimating lifetime costs

Since T1D patients are typically diagnosed at an early age compared to typical T2D patients and T1D patients therefore suffer from the disease for a longer portion of their life, lifetime costs are better for capturing the full burden of the disease and providing a fairer, more accurate, comparison between diseases. We use two approaches for measuring lifetime costs. First, we estimate the lifetime costs of a cohort of newly diagnosed patients by generating a longitudinal profile of costs from the cross-sections of patients in our sample stratified by current age and age of diagnosis. Second, using this longitudinal profile we estimate the amount incurred by the current population over the rest of its lifetime. The first approach seeks to measure the amount saved by a future cohort of newly diagnosed patients if onset of T1D could be prevented. The second method finds the savings by the current patient population if a cure (i.e., means to prevent or reverse the disease) were discovered today. These calculations admittedly neglect improvements in health care technology over time, but similar methods have been used to estimate the lifetime costs associated with smoking and obesity [20], [46].

To obtain expected expenditures, we divide the sample into seven age groups, starting with age group 3 to 18 (age 3 is the age of the youngest T1D patient in the sample). Sample size limitations prevented us from breaking down the 18 and under age group into smaller groups. We multiply total expenditures at each age by the probability of surviving to that age using published survival probabilities [44]. To obtain mortality rates of individuals with and without T1D, we use estimates from the literature on the relative mortality rates of diabetes patients vs. individuals without diabetes [42], [43]. Unfortunately, the relative death rates estimates from Gu et al. [42] do not distinguish between T1D and T2D. There is evidence that mortality rates are higher amongst patients with T2D, though early mortality rates are higher for T1D patients [47]. However, Cusick et al. [38] finds lower 5-year mortality rates amongst patients with T1D than patients with T2D, and Juutilainen et al. [48] find the risk of mortality from cardiovascular disease to be similar between T1D and T2D patients. As the results are inconclusive, we use figures from Gu et al. [42] since the data source is nationally representative data. However, although Gu et al. [42] note that mortality rates are higher for those patients with a longer history with diabetes, they do not report mortality rates for each age group by duration of diabetes, which would be an important area for future studies to examine. Furthermore, Gu et al. [42] do not provide mortality rate estimates for those under age 25. For mortality estimates of those under 25, we use figures from Dahlquist and Kallen [43] on the relative mortality children with T1D diagnosed between ages 0 to 14 compared with children without diabetes.

We calculate the present value of expected costs by discounting the stream of expenditures at a rate of three percent, as recommended by the World Health Organization [41].

Expected lifetime expenditures are described by
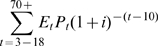
(6)where

*E*_t_ = expenditures during age interval if the individual survives through t
*P_t_* = probability of surviving through age interval
*t* = 10, 25, 35, 45, 55, 65, 70+
*i* = discount rate.

To estimate the size of the yearly cohort of new T1D patients, we use published T1D incidence rates and total population counts from the 2000 Census [4], [49]. We calculate the effect of preventing the disease by multiplying the number of new patients at each age by the present value of expected lifetime costs. Similarly, we compute the savings from a cure for T1D by multiplying the current number of T1D patients by the present value of expected lifetime costs. The difference between the present value of expected lifetime expenditures of patients with T1D and that of their matched counterparts is attributable to T1D.

## Results

### T1D population

Table 1 reports the number of unweighted and weighted observations classified as T1D using various cutoff ages for diagnosis. Note that this sample is only representative of the civilian noninstitutionalized population, leaving out patients with T1D in nursing homes or the military since MEPS does not collect information on these populations.

**Table 1:** T1D sample size by age of diagnosis.^a^

Age of diagnosis	N	Sample weighted total for average year (1999–2005)
30 or younger	563	756,377
35 or younger	632	855,697
40 or younger	721	966,238
45 or younger	817	1,089,755

a The patient is categorized with T1D if he reports being told by a physician that he has diabetes, is currently using insulin, and is diagnosed at or before age 45. We include patients under age 18 only if they are not considered obese. Patients diagnosed later than age 30 are only included if their BMI is 30 or lower.

Table 2 reports the size of MEPS sample. By combining data from 1999 (when the DCS began) to 2005 (the most recent year with available NHIS data), the total sample consists of 194,115 person-years. Using the MEPS sample weights, this represents 262 million non-institutionalized, civilians in an average year. Of the 11,071 that report having diabetes that have available data, 817 were diagnosed at or before age 45, are currently on insulin, and are not obese if they were diagnosed over the age of 30. The 817 person-years represent 1.1 million patients with T1D nationally in an average year between 1999 and 2005. The control group consists of those person-years where no diabetes (type 1 or type 2) is reported. The unweighted potential control group consists of 183,044 person-years. Table 3 shows the sample sizes by age of onset for each age group.

**Table 2:** Sample sizes, weighted and nonweighted.

Description	N	Sample weighted total for average year (1999–2005)
Person-years with MEPS & NHIS data	194,115	262 million
Answered ‘Yes’ to ever been diagnosed with diabetes	11,071	15.1 million
Categorized as T1D patient^a^	817	1.1 million
Potential control group (no diabetes diagnosis)	183,044	246 million

a The patient is categorized with T1D if he reports being told by a physician that he has diabetes, is currently using insulin, and is diagnosed at or before age 45. We include patients under age 18 only if they are not considered obese. Patients diagnosed later than age 30 are only included if their BMI is 30 or lower.

**Table 3:** Number of Type 1 Diabetes Patients by Current Age and Age of First Diagnosis (Sample Weighted Below).

	Current Age							
Age of Diagnosis	3–18	19–29	30–39	40–49	50–64	65–74	75+	Total
0–18	65	42	41	45	47	12	13	265
	71,036	57,253	60,994	64,154	76,812	13,813	18,643	362,706
19–29	-	50	63	56	65	14	9	257
		66,528	82,404	71,685	91,001	8,609	11,309	331,537
30–39	-	-	23	41	65	24	5	158
			38,747	60,093	86,567	29,740	7,075	222,222
40–45	-	-	-	14	76	20	27	137
				17,491	92,980	29,222	33,597	173,290
							**Total**	817
							**Sample Weighted**	1,089,755

*Note:* The patient is categorized with T1D if he reports being told by a physician that he has diabetes, is currently using insulin, and is diagnosed at or before age 45. We include patients under age 18 only if they are not considered obese. Patients diagnosed later than age 30 are only included if their BMI is 30 or lower.

Table 4 summarizes the results of the propensity matching assessment. The first three columns of Table 4 report the covariate means of key variables for the T1D group, the entire control group, and the matched control group. The average age of a T1D patient in the sample is 48 years and over half of the sample is female. Twenty percent of the patients under 18 with T1D also report having eczema. The bias is computed for the matched and unmatched control cases (fourth and fifth columns of Table 4). The last column of Table 4 reports the percentage reduction in bias between the matched and unmatched control cases. The bias reduction in propensity score is nearly 100 percent. In general, the bias for each covariate is greatly reduced by at least 80 percent. However, the matched cases do not match well on years spend in the US and birth weight, which applies for those under 18.

**Table 4:** Propensity score matching assessment – bias reduction from using a matched vs. unmatched sample.

Variable	T1D^a^ (N = 817)	Unmatched control^a^ (N = 183,044)	Matched control^a^ (N = 817)	Bias (Unmatched)	Bias (Matched)	Percent bias reduction
Propensity Score	0.20	0.09	0.20	100.1	0.0	100.0
Age (years)	48.44	34.80	47.84	70.0	3.5	95.0
Female	0.56	0.52	0.53	6.5	5.8	10.3
MSA	0.76	0.79	0.75	8.4	1.1	87.0
Non White	22.01	19.94	22.27	5.1	0.6	87.8
Northeast	0.14	0.16	0.15	3.5	0.7	80.1
Midwest	0.20	0.21	0.20	1.2	0.0	99.5
South	0.41	0.37	0.42	8.1	0.9	88.7
West	0.23	0.27	0.24	7.5	0.7	90.9
Year 1999	0.07	0.13	0.07	21.0	0.3	98.7
Year 2000	0.09	0.14	0.08	14.3	2.3	83.7
Year 2001	0.21	0.18	0.20	9.8	3.1	68.4
Year 2002	0.24	0.21	0.24	8.6	0.7	91.8
Year 2003	0.20	0.18	0.22	5.6	5.8	−2.3
Year 2004	0.19	0.18	0.18	2.8	1.7	38.8
Birth Weight (ozs)^b^	112.90	117.67	122.40	17.1	28.2	−64.8
Height (inches)	66.20	66.84	66.90	15.9	17.6	−10.5
U.S. Citizen	0.95	0.90	0.90	17.8	19.7	−10.6
In U.S.<15 Years	0.18	0.37	0.03	41.6	52.5	−26.3
Eczema^b^	0.20	0.06	0.20	40.7	0.0	100.0
Arthritis	0.34	0.12	0.28	51.7	11.0	78.8
Cancer	0.04	0.02	0.04	8.3	0.7	91.5
Mom Finished HS^b^	0.56	0.39	0.56	35.1	0.0	100.0
Dad Finished HS^b^	0.44	0.27	0.44	35.5	0.0	100.0

a All values are percents unless otherwise noted.

b Data available for those under 18 years of age only.

We examine the joint significance of the regressors in the logit estimation before and after matching. They are jointly significant before matching with an F-statistic of 19.3. When we rerun the estimation using just the T1D patients and the matched controls, we cannot reject the hypothesis that the covariates have no explanatory power, which indicates that the covariates are able to predict whether the individual has T1D. In other words, conditional on the propensity score, T1D is “randomly” assigned to either group.

To confirm the validity of T1D sample of 1.1 million patients in the MEPS, we apply the same T1D clinically-derived definitions that were described above to the NHANES data. We find a sample representing 1.4 million individuals nation-wide who have T1D, slightly larger than the MEPS sample. This figure is based on NHANES data for an average year between 1999 and 2004. The actual number of T1D patients is 140, weighted to represent 1.4 million persons.

NHANES collects information on fasting plasma glucose levels and c-peptide levels of a subset of their sample. A fasting plasma glucose level of 126 mg/dl or over indicates diabetes [32]. Although not universally agreed upon, a c-peptide test of 0.5 nmol/L or lower can be used to identify T1D patients [50]. Using these qualifications, the NHANES categorizes approximately 600,000 individuals with T1D nation-wide. However, only 400,000 of the patients categorized as T1D using our clinically-derived definitions had available plasma glucose and c-peptide test information. About 40 percent of the individuals defined as a T1D patient using our clinically-derived definitions who have available test data report a c-peptide test of less than 0.5 nmol/L and a plasma glucose of over 126 mg/dl, but fewer than 5 percent of T2D patients defined using our clinically-derived definitions display these test results (results available upon request). This information confirms that we are not likely leaving out many true T1D patients from our sample, but it is possible that we are including T2D patients in the sample.

### Medical costs

Table 5 reports medical expenditures attributed to T1D for hospital, ambulatory visits (office-based and outpatient), prescription drug (including medical supplies) and emergency room visits for an average year between 1999 and 2005 in 2005 dollars. The first column shows the mean yearly expenditure for patients with T1D by medical care type. All medical events in MEPS are self-reported. Therefore, it is possible that a hospital visit that started out as an emergency room visit was reported as simply a hospital visit and coded that way. MEPS does provide a variable that indicates whether a hospital visit began with an ER visit. However, we are unable to allocate those costs between the ER and hospital inpatient share of the visit. The second column lists medical expenditures by type of medical care for the matched control group of individuals without diabetes. The mean total yearly medical expenditures for the T1D group are nearly $10,000, and the expenditure for their matched counterparts is $3,580. The difference between the T1D group and the matched control group is the mean yearly value attributed to T1D and is $6,288 (4,426 to 8,150) in total medical expenditures. Expenditures on hospital visits, prescription drug and ambulatory visits are significantly different between the T1D and control group at the 1 percent significance level. Emergency room costs attributed to T1D are significantly different from zero at the 10 percent significance level.

**Table 5:** Mean yearly and total yearly medical expenditure for patients with T1D and matched controls (2005 dollars).

Outcome	T1D (1)	Matched control (2)	Attributed to T1D^a^ (1)–(2)	
Hospital Inpatient	3,506	956	2,550	(1,184–3,916)^***^
Ambulatory Visits	2,485	1,295	1,190	(470–1,909)^***^
Prescription Drug	3,089	823	2,266	(1,956–2,576)^***^
Emergency Room^b^	222	125	97	(−1–195)^*^
**Total Medical Expenditure (per capita)** c	**9,868**	**3,580**	**6,288**	(4,426–8,150)^***^

a Ninety-five percent confidence intervals in parentheses. ***, **, * indicate the 1 percent, 5 percent and 10 percent significance levels, respectively.

b ER visits are likely under-reported. It is possible that a hospital visit that started out as an emergency room visit was reported as simply a hospital visit, and coded that way. Although MEPS does provide a variable that indicates if a hospital visit began with an ER visit, we would not know how to allocate the costs between ER and hospital, so we do not use this variable.

c Outcomes do not add up to total because total medical expenditure includes some other factors (home health care, vision, etc.) that are each small.

While some patients with T1D may not experience any serious complications, complications that do arise can be quite severe and are likely to lead to more intensive medical care. We attempt to examine the medical use of patients with T1D with complications by using hospital nights as an indicator for complications. Of the 817 person-years in the T1D sample, 605 report zero hospital nights. Limiting the results to the 212 who do report at least one hospital night and comparing them with their matched counterparts yields results shown in Table 6. Average medical expenditures are substantially higher for the limited sample that report at least one hospital night. Patients with T1D with at least one hospital night spend $25,858 in total medical expenditures. The matched control spend $2,720. Average yearly total medical expenditure attributed to T1D then jumps to $23,137 (17,288 to 28,986) per year.

**Table 6:** Mean yearly medical expenditure with at least one hospital night (2005 dollars).

Outcome	T1D (1)	Matched control (2)	Attributed to T1D^a^ (1)–(2)	
Hospital Inpatient	15,019	788	14,231	(10,013–18,449)^***^
Ambulatory Visits	5,793	772	5,021	(2,236–7,806)^***^
Prescription Drug	3,797	787	3,010	(2,257–3,763)^***^
Emergency Room^b^	544	98	446	(187–705)^***^
**Total Medical Expenditure (per capita)** b	**25,858**	**2,720**	**23,137**	**(17,288**–28,986)^***^

a Ninety-five percent confidence intervals in parentheses. ***, **, * indicate the 1 percent, 5 percent and 10 percent significance levels, respectively.

b Outcomes do not add up to total because total medical expenditure includes some other factors (home health care, vision, etc.) that are each small.

### Indirect costs

Indirect costs for patients with T1D and their matched counterparts during an average year are listed in Table 7. Work days and bed days are significantly different for the two groups. Patients with T1D miss on average 5.5 (3.2 to 8.4) more days than individuals without diabetes per year and experience 7.6 more bed days. Bed days may include lost work days, so the two should not be added. T1D patients under 18 miss 3.3 (2.1 to 4.0) more school days. Individual income (individual income is compared only for those over 18 years of age) for patients with T1D is $7,164 (9,268 to 6,008) lower than income of individuals without diabetes, and household income is $9,480 (5,701 to 11,034) lower for T1D patients.

**Table 7:** Mean yearly indirect costs for patients with T1D and matched controls (2005 dollars).

Outcome	T1D (1)	Matched Control (2)	Attributed to T1D (1)–(2)^a^	
Missed work days	10.8	5.3	5.5	(3.2–8.4)^***^
Bed days	11.2	3.6	7.6	(6.4–9.5)^***^
Missed school days	3.7	0.4	3.3	(2.1–4.0)^***^
High school graduate (%)	47.7	48.1	−0.4	(−4.0–3.1)
College graduate (%)	14.4	12.7	1.7	(−4.7–4.1)
Graduate education (%)	13.2	13.5	−0.3	(−2.7–2.1)
Individual income^b^	22,958	30,122	−7,164	(−9,268–−6,008)^***^
Household income	45,511	54,991	−9,480	(−11,034–−5,701)^***^

a *** indicates significance at the 1 percent level, 95 percent confidence intervals in parentheses.

b Individual income is estimated for those over 18. Where applicable, income is split evenly between spouses.

### Lifetime costs attributed to T1D

Table 8 reports average yearly medical expenditures attributed to T1D by age group. On average, an individual aged 3 through 18 incurs an additional $1,981 (−918 to 4,880) in medical expenditures per year due to T1D. However, this figure is not significant. T1D attributable expenditures rise for the most part to a peak in the 65 to 74 age group and decrease slightly after that. At older ages, nondiabetes related medical conditions may be the main driver of medical expenditures, especially if those who survive to this tend to be healthier.

**Table 8:** Age distribution of medical costs attributed to T1D.

Age group	N	Sample weighted	Avg. yearly T1D medical cost^a^	
3–18	65	71,036	$1,981	(−918–4,880)
19–29	92	123,782	$3,573	(145–7,001)^**^
30–39	127	182,145	$6,471	(−443–13,386)^*^
40–49	156	213,423	$4,999	(1,661–8,337)^***^
50–64	253	347,360	$7,079	(4,239–9,919)^***^
65–74	70	81,385	$10,100	(3,105–17,095)^***^
75+	54	70,624	$8,709	(4,767–12,651)^***^

a Ninety-five percent confidence intervals in parentheses. ***, **, * indicate the 1 percent, 5 percent and 10 percent significance levels, respectively.

Table 9 reports the expected lifetime medical cost and income loss attributable to T1D by age of onset for a new cohort of T1D patients. We estimate the size of the cohort of new T1D patients to be 28,430 in an average year between 1999 and 2005, which is consistent with various other findings [48]. The largest number of new patients falls within the 10 to 19 age group, which consequently faces the greatest share of expected lifetime expenditures due to T1D. The present value of expected medical and indirect costs for the whole cohort of new T1D individuals is $3.3 billion (2.1 billion to 4.5 billion) and $7.3 billion (4.5 billion to 10.1 billion), respectively.

**Table 9:** Expected lifetime medical and indirect costs attributed to T1D by a new cohort of patients (2005 dollars).^a^

Age of onset	Number of new patients	Medical (millions)		Income loss^b^ (millions)	
3–9	6,483	$746	(443–1,069)^***^	$1,208	(667–1,749)^***^
10–19	11,980	$1,489	(807–2,171)^***^	$2,923	(1,604–4,242)^***^
20–29	3,528	$337	(193–481)^***^	$1,130	(617–1,643)^***^
30–39	3,976	$395	(240–550)^***^	$1,279	(841–1,717)^***^
40–45	2,464	$309	(128–491)^***^	$776	(402–1,150)^***^
**Total**	**28,430**	**$3,276**	**(2,098**–**4,454)** ^***^	**$7,316**	**(4,513**–**10,119)** ^***^

a Ninety-five percent confidence intervals in parentheses. ***, **, * indicate the 1 percent, 5 percent and 10 percent significance levels, respectively.

b Income is compared only for those over 18 years of age, but discounted back to the age of diagnosis.

Table 10 shows the expected lifetime medical and indirect costs by age for the current T1D patients in the sample. The present value of expected lifetime costs attributed to the disease for the 1.1 million T1D patients estimated in an average year between 1999 and 2005 was $133.7 billion (97.0 billion to 170.4 billion) in medical expenditures and $289.2 billion (211.7 billion to 366.7 billion) in lost income.

**Table 10:** Expected lifetime medical and indirect costs attributed to T1D by current patients (2005 dollars).^a^

Age	Number of current patients	Medical (millions)		Income loss^b^ (millions)	
3–9	18,543	$3,799	(2,274–5,324)^***^	$5,069	(−3,312–13,450)
10–19	61,642	$7,158	(4,343–9,973)^***^	$11,409	(4,086–18,732)^***^
20–29	114,633	$19,146	(11,620–26,672)^***^	$39,085	(25,363–52,807)^***^
30–39	182,145	$27,760	(19,254–36,266)^***^	$69,029	(51,428–86,630)^***^
40+	712,792	$75,832	(51,871–99,793)^***^	$164,624	(112,751–216,497)^***^
**Total**	**1,089,755**	**$133,695**	**(97,015**–**170,374)** ^***^	**$289,216**	**(211,727**–**366,705)** ^***^

a Ninety-five percent confidence intervals in parentheses. ***, **, * indicate the 1 percent, 5 percent and 10 percent significance levels, respectively.

b Income is compared only for those over 18 years of age, but discounted back to the current age.

## Discussion

The CDC finding that T1D accounts for 5 to 10 percent of all diabetes cases remains a popular benchmark [3]. Due to an aging population, increases in the prevalence of obesity and improvements in detection, the number of T2D patients has been rapidly growing [2], [7]. Although some studies have shown that the number of patients with T1D is increasing as well [4], there is no obvious reason to believe that rate of increase between the two major types of diabetes should be related. With the rapid growth in the prevalence of T2D, using the commonly cited 5 to 10 percent benchmark can lead to widely varying figures on the number of T1D cases. For example, The Juvenile Diabetes Research Foundation International (JDRF) lists as high as 3 million patients with T1D as a figure on its website [51]. We identify 1.1 million T1D patients, which falls at the lower end of the 5 to 10 percentage range of the total number of individuals with diabetes reported by the ADA and CDC. According to the CDC, 5 to 10 percent of the number of total individuals with diabetes per year in 1999 through 2005, was 790,000 to 1.6 million T1D patients [52]. Dall et al. [13], using different data and ICD-9-CM diagnosis codes to identify T1D patients, also find a T1D patient population of 1 million, similar to our estimate of 1.1 million T1D patients.

### Medical Costs

We find total yearly medical expenditure attributable to T1D to be $6.9 billion (5.9 billion to 7.9 billion) over the medical expenditures of a matched group of nondiabetic individuals (see Table 11). Per capita expenditures amount to $6,288 (4,426 to 8,150) per year where hospital inpatient visits and prescription drugs including medical supplies account for over 75 percent of the yearly cost attributable to T1D.

**Table 11:** Summary of estimated costs attributable to T1D (2005 dollars).^a^

Cost component	Per capita costs		Total yearly costs^c^ (in billions)		Lifetime costs^d^			
					Newly diagnosed cohort of 30,000 (in billions)		Currently diagnosed 1.1 million patients (in billions)	
Medical	6,288	(4,426–8,150)	6.9	(5.9–7.9)	3.3	(2.1–4.5)	133.7	(97.0–170.4)
Indirect^b^	7,164	(6,008–9,268)	7.5	(4.6–10.2)	7.3	(4.5–10.1)	289.2	(211.7–366.7)
**Total**	**13,452**	**(9,193–17,711)**	**14.4**	**(11.5–17.3)**	**10.6**	**(7.2–14.0)**	**422.9**	**(327.2–519.4)**

a Ninety-five percent confidence intervals are in parentheses.

b Indirect costs are comprised of lost income for those 18 and over.

c Total yearly costs are estimated for the 1.1 million T1D patients.

d Lifetime costs are the discounted present value of the expected stream of costs over the lifetime.

We report mean values, but the underlying population is heterogeneous in terms of costs. There is a small subpopulation of T1D patients who rarely experience any complications associated with the disease. They are currently the focus of scientific studies, though a common factor has not yet been found [53]. At the other end of the spectrum, we use those patients who report at least one hospital night in the past year to proxy for patients with complications. We find that this small subpopulation spends on average over $23,000 year, or nearly 3.5 times the mean value, on medical care attributable to T1D, mostly attributable to the fourfold increase in hospital inpatient expenditures.

We compare our figures to the ADA papers, noting that our methodology differs from that of the ADA [6], [2]. Removing nursing home and hospice expenses from the ADA studies' overall diabetes medical cost (since MEPS only samples the non-institutionalized population), the ADA reports $77.4 billion (2002 dollars) and $109 billion (2007 dollars) in medical expenditures attributed to diabetes. (Note that there are differences in methodology, as well as data sources, between the ADA 2003 [6] and ADA 2008 [2] papers.) Our estimate of $6.9 billion in medical expenditures falls within the 5 to 10 percent range of overall diabetes medical expenditures (see Table 12) and is roughly in proportion to the population factor. In comparison with Dall et al. [13], our estimate of medical costs is lower than the $10.5 billion the authors estimate for 2007. However, of the $10.5, $4.4 billion was found to be attributed to nursing or residential facility care. Removing this component, our estimates of medical costs attributed to T1D are similar to those found by Dall et al.

**Table 12:** Comparison of yearly costs: T1D vs. relevant ADA all diabetes estimates (2005 dollars).

Cost component	T1D (billions)	ADA – all diabetes (billions)^a^	T1D percent of total
Medical	6.9	101.9^b^	6.8%
Indirect	7.5	29.3^c^	25.6%

a We used a conservative rate of inflation of 3% per year for 2006 and 2007 from the BLS to deflate the ADA figures.

b We removed nursing home and hospice costs from the figure since the MEPS does not collect information on the institutionalized population.

c This figure was obtained by removing the $26.9 billion attributed to early mortality from the total of $58.2 billion in indirect costs because our methodology concerning early mortality differed significantly. If we left early mortality costs in both the ADA and our calculations, indirect costs for T1D would be 13.9 percent of costs for all diabetes.

Per capita total medical expenditures by people with diabetes are generally comparable between our study and the ADA papers. However, the matched control group of individuals without diabetes in our analysis spend less on medical care than the age-sex adjusted individuals without diabetes in the ADA paper ($3,580 (2005 dollars) in our paper compared with $4,464 (2007 dollars) in the ADA paper). We use a matching technique to match individuals without diabetes as closely as possible to T1D patients based on a number of factors. Therefore, our T1D control group may in general be healthier than the age-sex adjusted control group used in the ADA papers (recall that we condition on being nonobese for a portion of our sample), indicating the costs we estimate are a lower bound of the cost attributed to T1D.

### Lost Income

Total income loss estimated for the 1.1 million T1D patients in our sample for an average year between 1999 and 2005 is $7.5 billion (4.6 billion to 10.2 billion) in 2005 dollars, as shown in Table 11. This figure includes early mortality costs that are estimated to be $3.0 billion for T1D patients and $2.9 billion for those without diabetes. Thus, $120 million is attributed to early mortality from T1D. Individuals without diabetes are estimated to have higher incomes than patients with the disease. If those without diabetes were to have exactly the same earnings profile as a T1D patient, the early mortality costs attributed to the disease would be estimated to be $1.7 billion.

T1D individuals miss 5.5 (3.2 to 8.4) more work days, experience 7.6 (6.4 to 9.5) more bed days, and miss 3.3 (2.1 to 4.0) more school days per year than their matched counterparts. We do not find that educational attainment is significantly affected by T1D. These findings are consistent with other published findings [15].

The ADA papers measure indirect costs by estimating and valuing lost productivity through lost work days and bed days, disability, and increased mortality [2], [7]. The 2008 paper adds presenteeism (reduced performance when at work), which is calculated using values from the literature. See ADA, 2008 [2] page 8 for more information. After combing the literature, the ADA uses a productivity loss associated with diabetes (all types) of 14 days per worker per year. Our use of income loss theoretically encompasses lost productivity due to disability, lost work days and bed days. Presenteeism would be included insomuch as the decreased productivity is reflected in salaries.

The per capita figure for the number of lost work and bed days reported by the ADA is similar to our findings. The ADA finds total indirect costs of $58 billion in 2007 dollars. Our estimate of $7.5 billion (4.6 billion to 10.2 billion) in income loss is over 10 percent of the ADA estimation of indirect total diabetes costs. However, mortality costs in our study are not directly comparable to the ADA number due to important differences in methodologies. The ADA studies do not account for the possibility of early mortality for those individuals without diabetes. Removing mortality costs from both figures, we find that non-mortality related indirect costs of the T1D population make up over 25 percent of the total diabetes costs (Table 12). Thus, the indirect costs of T1D make up a disproportionately higher percentage of the overall diabetes costs than the population factor would suggest. Therefore, we find that it is inadequate to assume that the yearly cost of T1D is simply the same proportion of total costs as the number of type 1 diabetic patients compared with the total number of patients with diabetes for indirect costs.

Dall et al. [13] estimated indirect costs by calculating the proportion of days spent receiving medical services related to T1D by age and gender, where office and outpatient visits accounted for half days and emergency room and hospital visits accounted for full days. The authors estimated the long-term disability costs associated with T1D by using the proportion of hospital inpatient days. The authors estimate that $4.3 billion in nonmedical costs were attributed to T1D in 2007. The lower figure compared with our findings reflect the differing methodologies between the two studies.

### Lifetime costs

It is not surprising that indirect costs make up a large portion of total costs of T1D, given the typical early onset of the disease. Once on a lower earnings trajectory, it may not be possible to make up for the income difference; instead the differences may become even more severe as time passes. The calculation of lifetime costs of T1D, presented here for the first time, is particularly relevant for a chronic, childhood disease such as T1D, where costs are incurred over a long lifetime. Expected discounted lifetime income losses of a new cohort of 30,000 T1D are estimated to be $7.3 billion (4.5 billion to 10.1 billion). The expected discounted lifetime medical costs patients of a new cohort of 30,000 T1D are $3.3 billion (2.1 billion to 4.5 billion). For the current 1.1 million T1D patients, the present value of their expected lifetime medical and indirect costs is $133.7 billion (97.0 billion to 170.4 billion) and $289.2 (211.7 billion to 366.7 billion), respectively.

Lifetime costs for several diseases associated with obesity are estimated by Thompson et al. [46]. The authors calculate lifetime medical costs attributed to obesity beginning at age 35 by BMI. The authors focused on five diseases related to obesity: hypertension, hypercholesterolemia, T2D, coronary heart disease, and stroke. The discounted expected lifetime medical costs range from $20,000 to $39,000 per person in 2005 dollars (inflated from 1996 dollars) depending upon BMI and gender. If these costs were discounted to birth, they would be significantly smaller. This would, however, be offset by whatever costs are incurred due to obesity in earlier ages. We estimate the cost to a newly diagnosed T1D patient that is age 14 to be $130,000 over his lifetime in medical costs, which is considerably larger. For a newly diagnosed 35-year old with T1D, his expected rest-of-lifetime medical cost attributable to the disease is $100,000. Although the methodologies in the studies differ, it is clear that lifetime costs of T1D are substantial.

### Conclusions

Until now, the economic costs specific to T1D have not been adequately measured. Together with the recent work of Dall et al. [13], and employing a complementary methodology, this research is the first nationally representative cost of illness study for T1D including lifetime costs. Our estimates show that patients with T1D incur a disproportionately large indirect costs burden as a result of their disease when compared with patients with T1D.

It is important to note that our analysis is likely to provide an underestimate of the true societal costs of T1D. Due to data limitations, we necessarily omitted costs such as nursing home expenditures and quality-of-life effects, which may be substantial. Dall et al. [13] find the costs of institutional care to be $4.4 billion, a substantial portion of the total $10.5 billion the authors estimate to be the cost of T1D. The use of income as a measure of indirect costs is also an imperfect measure of productivity. Including information on the type of diabetes in future waves of national health studies would help improve future research on the economic costs of this patient population. Comparison of lifetime costs for T2D and other chronic diseases in future economic studies would enhance our understanding of the economic impact of the diseases on individuals and society. However, using propensity score matching methods may not be as effective with T2D patients, since lifestyle behaviors not easily captured in survey data affect the probability of having the disease.

While continued work is required to better understand the burden of T1D, COI studies can be used by policy makers, researchers, advocates, doctors, patients to assess the economic impact of the diseases on the individual and society. Used in conjunction with more traditional biomedical research studies, COI studies allow a more complete picture of the impacts of a particular disease to be developed. They can add to a thorough understanding of the costs and benefits of a particular disease treatment or to a better comparison among various diseases. This study is a crucial step forward in capturing the full costs, both yearly and over a lifetime, of T1D. We have shown that the disease has a substantial and disproportionate economic impact, particularly in the indirect costs. Over their lifetime, a patient with T1D incurs substantial medical and indirect costs as a result of their disease, understanding these costs is a crucial component of understanding the full impact of the disease.
